# MicroRNA Alteration, Application as Biomarkers, and Therapeutic Approaches in Neurodegenerative Diseases

**DOI:** 10.3390/ijms23094718

**Published:** 2022-04-25

**Authors:** T. P. Nhung Nguyen, Mandeep Kumar, Ernesto Fedele, Giambattista Bonanno, Tiziana Bonifacino

**Affiliations:** 1 Pharmacology and Toxicology Unit, Department of Pharmacy, University of Genoa, Viale Cembrano 4, 16148 Genoa, Italy; nguyen@difar.unige.it (T.P.N.N.); kumar@difar.unige.it (M.K.); bonanno@difar.unige.it (G.B.); bonifacino@difar.unige.it (T.B.); 2 IRCCS Ospedale Policlinico San Martino, 16132 Genoa, Italy; 3 Inter-University Center for the Promotion of the 3Rs Principles in Teaching & Research (Centro 3R), 56122 Genoa, Italy

**Keywords:** microRNAs (miRNAs), neurodegenerative diseases, Alzheimer’s disease (AD), amyotrophic lateral sclerosis (ALS), Parkinson’s disease (PD), multiple sclerosis (MS), Huntington’s disease (HD), dysregulation, biomarker

## Abstract

MicroRNAs (miRNAs) are essential post-transcriptional gene regulators involved in various neuronal and non-neuronal cell functions and play a key role in pathological conditions. Numerous studies have demonstrated that miRNAs are dysregulated in major neurodegenerative diseases, such as Alzheimer’s disease, Parkinson’s disease, multiple sclerosis, amyotrophic lateral sclerosis, or Huntington’s disease. Hence, in the present work, we constructed a comprehensive overview of individual microRNA alterations in various models of the above neurodegenerative diseases. We also provided evidence of miRNAs as promising biomarkers for prognostic and diagnostic approaches. In addition, we summarized data from the literature about miRNA-based therapeutic applications via inhibiting or promoting miRNA expression. We finally identified the overlapping miRNA signature across the diseases, including miR-128, miR-140-5p, miR-206, miR-326, and miR-155, associated with multiple etiological cellular mechanisms. However, it remains to be established whether and to what extent miRNA-based therapies could be safely exploited in the future as effective symptomatic or disease-modifying approaches in the different human neurodegenerative disorders.

## 1. Introduction

**Figure 1:**
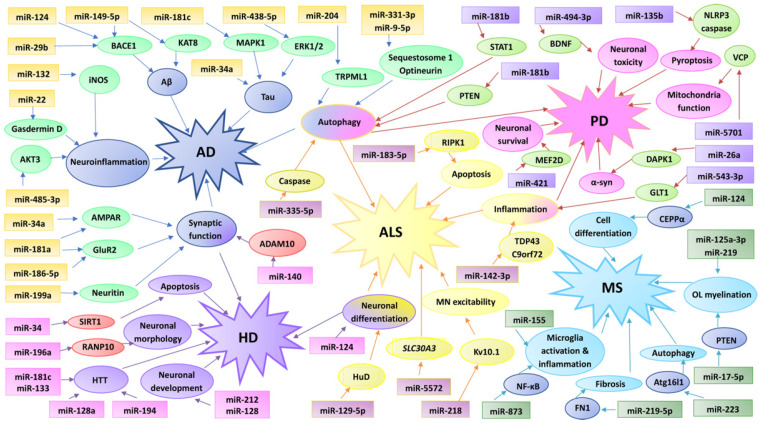
Complexity of the links between different miRNAs and molecular pathways–targets involved in neurodegenerative diseases.

MicroRNAs (miRNAs) are small, single-stranded RNA molecules of 20–23 nucleotides that do not encode a protein [1,2,3,4]; instead, they operate by binding to the 3′-untranslated region (3′-UTR) of mRNA to inhibit target expression [1,2]. Studies have shown that miRNAs play crucial roles in regulating a wide variety of biological processes, such as stress, cell fate, morphogenesis, synaptic plasticity, apoptosis, mRNA splicing, deoxyribonucleic acid (DNA) methylation, circadian rhythms, angiogenesis, cell cycle, endocrinological regulation, immunomodulation, and neuroprotection, and are dysregulated in many central nervous system (CNS) diseases (Figure 1) [3]. The neurological-/neurodegenerative-disorder-linked miRNA activity in the CNS has gained an increasingly significant role in recent years [1,2,3,4].

Neurodegenerative diseases (NDs) affect millions of people worldwide, causing significant societal, emotional, and economic burdens [4,5]. Most NDs are based on multicomplex pathological mechanisms. Due to the impact of NDs on human health and the lack of definitive therapies for almost all of them, early detection before disease onset and effective therapeutic interventions can helpfully reduce cost and time efforts. Thus, scientists investigated miRNAs as sensitive diagnostic and prognostic biomarkers [4], and miRNA-based therapeutic approaches by regulating miRNA expressions via miRNA activity enhancement (miRNA mimics or agomirs) or inhibition (miRNA inhibitors or antagomirs) were also analysed [5].

This article aims to provide a comprehensive overview of miRNA alterations in NDs, their contribution as potential biomarkers, and possible therapeutic applications. To this purpose, we evaluated the most recent studies related to miRNA dysregulations in ND, the pathogenic pathways in vitro and in vivo in animal models and humans, the promising miRNA role as biomarkers, the novel miRNA-based therapies, the delivery to CNS techniques, and their advantages and limitations. Finally, we identified the cross-over of some miRNAs among different NDs.

## 2. Biology of miRNAs

Since the first miRNAs, *lin-4* and *let-7*, were discovered in *Caenorhabditis elegans* in 1993, over 2000 miRNAs have been to date reported on http://www.mirbase.org (accessed on 22 February 2022) [6,7]. Due to the number of miRNAs, 30–80% of the human genes are possibly under miRNA regulation [2,8]. Each miRNA can interfere with multiple functions of a single cell type, and several miRNAs can interact to target the same mRNA [1]. Most miRNAs are located in the intronic gene portion, whereas others are localized in the coding position [8]. Furthermore, numerous investigations have shown that miRNA expression differs between tissues and cell lines [4]. Therefore, the interaction between a given miRNA and its target genes depends on many factors, such as the miRNA’s location, miRNA–mRNA quantities, and affinity [2]. miRNAs are assumed to have critical roles in many biological processes in physiological and pathological conditions [1,2]. Indeed, miRNA dysregulation has been associated with several neurological disorders [1]. In addition, both mature miRNAs and their precursors are secreted into extracellular fluids; thus, they can be considered signaling molecules for cell-to-cell communication or potential biomarkers for various diseases [2,8].

The miRNA biogenesis process generally starts with the miRNA gene co-operating post- or cotranscriptionally with RNA polymerase II/III transcripts, and this pathway includes canonical and noncanonical branches [2]. In the dominant canonical pathway, the miRNA primary transcripts (pri-miRNAs) are transcribed from their genes in the nucleus [8]. Pri-miRNAs transform into miRNA precursors (pre-miRNAs) under the action of the complex ribonuclease III enzyme Drosha and its cofactor DiGeorge Syndrome Critical Region 8 (DGCR8), an RNA binding protein [5,9]. Then, pre-miRNAs are exported to the cytoplasm via exportin-5/Ras-related nuclear protein-GTPase (XPO5/Ran-GTP) complex. Here, the extended miRNA duplex is created under the effect of a Dicer protein, an RNase III endonuclease. After that, one of the duplex strands recruits the Argonaute 2 (AGO2) protein to form a mature RNA-induced silencing complex (miRISC) to join either the total complementarity or the partial complementarity pathway that binds the 3′-UTR of the mRNA target, leading to its degradation or translation repression, respectively [5,8,10,11]. Some miRNAs, known as noncanonical miRNAs, are generated by different biogenesis pathways that can be grouped into Drosha-/DGCR8-independent and Dicer-independent pathways [5]. Sources of noncanonical miRNAs include Dicer-independent miRNAs, mirtrons, small nucleolar RNA-derived miRNAs, and tRNA-derived miRNAs [5]. For example, short-hairpin RNA is initially cleaved in the nucleus by the microprocessor complex consisting of DGCR8–Drosha. Later, this is exported to the cytoplasm via XPO5/Ran-GTP and further processed via AGO2-dependent, but Dicer-independent, cleavage [2]. In both pathways, a functional miRISC complex is created, which binds to the targeted mRNAs to suppress its expression [2,11].

miRNAs can regulate several gene expressions due to the miRNA–mRNA interaction [2,3]. The specific binding site of miRNA is at the 3′-UTR of its target mRNA, resulting in mRNA deadenylation and decapping. However, other miRNA binding sites include the 5′-UTR, coding sequences, and the promoter regions [2]. Most studies revealed that miRNAs inhibit gene expression via miRISC [2,3]. However, it has also been reported that miRNAs can induce gene upregulation under some circumstances, as in quiescent mammalian cells and immature oocytes, involving AGO2 and Fragile-X-mental-retardation-syndrome-related protein 1a (FXR1) [12].

miRNAs are mainly regulated at both transcriptional and post-transcriptional levels in the nucleus. For example, the transcriptional repressor element 1 silencing transcription factor, when activated, led to miR-132 silencing in the hippocampal CA1 neurons in an in vivo model of ischemic stroke [13]. The pituitary homeobox 3 transcription factor and miR-133b form a negative feedback loop influencing the differentiation of the midbrain dopamine neurons [14]. Meanwhile, the post-transcriptional pathways can affect the pri- or pre-miRNA stability or processing via the miRNA biogenesis enzymes, such as Dicer and Drosha [15,16,17]. These miRNA biogenesis proteins can be involved in the pathogenesis of several diseases, including the NDs. In a PD mouse model, inhibiting c-jun N-terminal kinase (JNK)-mediated microglial Dicer rescued neuroinflammation and reduced neuronal loss [15]. Controlling the Dicer complexity level involving the stress granule pathway by enoxacin gave benefits in two ALS mouse models [16]. TAR DNA-binding protein 43 (TDP-43), a key protein in ALS, interacted with the nuclear Drosha complex and bound to the pri-miRNA directly; it also bound with the Dicer complex to the loops of pre-miRNAs in the cytoplasm [17].

## 3. Alzheimer’s Disease

Alzheimer’s disease (AD) is a progressive brain disorder leading to a severe cognitive decline [18] that hopefully can be ameliorated by several new compounds [19,20,21]. The prevalence of AD increases substantially with age in both genders, and it will affect around 107 million people worldwide by 2050 [22]. The molecular and biological pathways of AD etiopathology are still not not fully understood. However, leading mechanisms include the accumulation of beta-amyloid (Aβ) plaques and neurofibrillary tangles due to hyperphosphorylation of Tau that are associated with gliosis, neuronal loss, cerebrovascular amyloidosis, oxidative stress, inflammation, and significant synaptic changes [23,24,25,26,27].

In normal conditions, Aβ is generated in neurons and released to the extracellular space, where it becomes a target of microglia and astrocytes for degradation. Initially, in the brain, the large molecule amyloid precursor protein (APP) can be cleaved under the action of β-secretases, with BACE1 being the major β-secretase species, to form Aβ40 and Aβ42 [23]. Soluble Aβ40 is more abundant than Aβ42; however, Aβ42 has a higher propensity for aggregation to generate amyloid plaques that show neurotoxic effects in AD [27]. Tau is a microtubule-associated protein that contributes to microtubule stability and its hyperphosphorylation is present in the brain of AD patients [24]. Furthermore, this hyperphosphorylation causes Tau detachment from microtubules and subsequent microtubule instability, self-aggregation, and neurofibrillary tangle formation. Many protein kinases and phosphatases regulate the phosphorylation status of Tau in phosphorylation-site-dependent manners [24,27]. In addition, the acetylation of Tau (Ac-Tau) promotes Tau aggregation, which suggests that Ac-Tau plays a role in Tau’s pathologic transformation [24,27]. Besides, the detrimental effects may come from the synergistic interaction between Aβ and Tau that triggers neurodegeneration in AD [24,26].

Among the complex multifactorial mechanisms, miRNA alterations may have a role in AD pathogenesis [27,28]. Consequently, miRNAs have been considered potential biomarkers and therapeutic agents in counteracting the disease [28,29].

### 3.1. miRNA Pathological Traits in Alzheimer’s Disease

Several specific miRNAs have been implicated in AD pathogenesis and they are involved in the following molecular mechanisms:

i Regulation of Aβ deposition (upregulation of miR-149-5p [30], miR-128 [31], and miR-126 [32]; downregulation of miR-520c [33,34], miR-124 [35], miR-101 [29], miR-107 [29,36], miR-328 [33,37], miR-29 and miR-29a/b-1 [33], miR-298 [33], miR-16 [33,38], miR-17 [33,39], miR-9 [33], miR-195 [33,40], miR-106 [33,34], miR-15b [41], and miR-132-3p [42]; mixed regulation: miR-125b [43,44,45]);
ii Hyperphosphorylated Tau protein accumulation (upregulation of miR-483-5p [46], miR-181c-5p [47]; miR-125b [33], miR-26b [48], miR-199a [49], miR-34a [33], miR-146, and miR-146a [33]; downregulation of miR-106b [33,50], miR-15a [33,51], miR-101 [33], miR-512 [33,52], and miR-132/-212 [33,53]);
iii Synaptic dysfunction (upregulation of miR-181a [54], miR-186-5p [55,56], miR-26b [48], miR-30b [33], miR-124 [33], miR-574 [33], miR-206 [33], miR-142-5p [33], miR-34a [57], and miR-199a [49]; downregulation of miR-10a [33] and miR-188-5p [33]);
iv Neuroinflammation (upregulation of miR-485-3p [58], miR-206 [33], miR-32-5p [33], miR-155 [33,59], miR-125b [33], and miR-146a [33]; downregulation of miR-132 [60], miR-22 [61], miR-331-3p [62], miR-26a [29], miR-29a [33], and miR-let-7a [33]);
v Autophagic dysfunction (downregulation of miR-204 [63], miR-214-3p [33], miR-299-5p [33], miR-132/212 [33,53], miR-331-3p [64], and miR-9-5p [64]).

As to the Aβ synthesis pathway, miR-124, miR-29, and miR-149-5p participate in β-site amyloid precursor protein cleaving enzyme (BACE) activity by directly targeting the 3′-UTR position and by regulating APP expression [30,35,65,66,67]. In the PC12 cellular AD model, miR-124 mimic or inhibitor could increase or decrease BACE1 expression, a key enzyme of APPβ generation, and a miR-124 inhibitor also increased the number of necrotic and apoptotic cells in vitro [35]. Moreover, miR-149-5p levels increased and Lysine acetyltransferase 8 (KAT8), a direct target of miR-149-5p, decreased in plasma of AD patients [30]. In the AD 293/APPsw cell model, miR-149-5p inhibition upregulated the expression of KAT8 and H4K16ac, an epigenetic modification of the DNA-packaging Histone H4, and displayed neuroprotective effects [30]. In summary, the inhibition of miR-149-5p delivery leads to BACE downregulation and upregulation of BACE2, a BACE1 homolog that antagonizes BACE1 and blocks Aβ production [30].

Antagomir of miR-15b decreased the apoptosis of Aβ-treated SH-SY5Y cells and its mimic reduced BACE1 level in HEK293 cells [41]. Overexpression of miR-29 (miR-29a, miR-29b) downregulated their gene targets, BACE1 and BIM, in the transfected HEK-293T cells [65]. Moreover, injection of miR-29b-containing exosomes in the hippocampal CA1 region rescued the spatial learning and memory impairments in an AD rat model [65]. Meanwhile, the other family member of miR-29 (miR-29c) not only directly regulated BACE1 expression in HEK-293 cell lines and in the APPswe/PSΔE9 mice [68], but also targeted the neuron navigator 3 (an axon guidance regulator) in the same transgenic AD mouse model [66]. Interestingly, miR-125b also regulated multiple targets, although it showed different types of regulation [43,44,45]. Overexpression of miR-125b in Neuro2a APPSwe/Δ9 cells increased APP, BACE1, Aβ, and Tau levels, enhanced inflammatory factors, and suppressed Sphingosine kinase 1, which can modulate different processes such as cell death/survival and learning and memory formation [44]. On the contrary, overexpression of miR-125b-5p attenuated Aβ toxicity in Aβ-treated N2a cells via targeting BACE1 [43]. miR-107 was supposed to have several targets, including BACE1, fibroblast growth factor 7 (a proliferation, inflammation, and apoptosis mediator), and cyclin-dependent kinase 5 regulatory subunit 1 (a regulator of brain development and function) [36,69,70]. miR-107 reduction correlated with the increase in BACE1 during AD progression in humans [36]. Similarly, miR-132-3p directly targeted BACE1 or histone deacetylase 3 that played a critical role in cognitive impairment [42,71]. Overexpression of miR-132-3p reduced apoptosis in Aβ42-treated SH-SY5Y cells and alleviated memory impairments in AD rats via modulating BACE1 [42].

Moreover, miR-181c could directly bind *LINC00507*, a long noncoding RNA upregulated in the hippocampus and cerebral cortex of APP/PS1 mice and Aβ42-transfected SH-SY5Y cells. On the other side, *LINC00507* regulates the expression of microtubule-associated protein Tau (MAPT) and Tau-tubulin kinase-1 (TTBK1), whose genes are a direct target of miR-181c-5p. *LINC00507* also mediates Tau protein hyperphosphorylation by activating the P25/P35/GSK3β signaling pathway through regulating MAPT/TTBK1 by sponging miR-181c-5p, which induces Tau hyperphosphorylation in AD [47]. miR-438-5p bound to the extracellular signal-regulated kinases 1 and 2 in HEK293 cell overexpressing Tau, thus leading to the reduction of phosphorylated Tau [46,72].

Many miRNA targets, such as synaptic α-amino-3-hydroxy-5-methylisoxazole-4-propionic acid receptors (AMPAR), the cyclic adenosine monophosphate response element-binding protein (CREB1), sirtuin1 (SIRT1), and the methyl CpG-binding protein 2 (MECP2), are involved in synaptic plasticity [29]. In 3xTg-AD mouse hippocampal synaptosomes, miR-181a negatively modulated synaptic plasticity via AMPA receptors, affecting the glutamate GluA1 and GluA2 subunits without rescuing translin, an miRNA-regulating protein [54]. miR-181a regulated other plasticity-related proteins, including GluA2, CREB1, SIRT1, cFos, Ca^2+^/calmodulin-dependent protein kinase II, and protein kinase AMP-activated catalytic subunit alpha 1. Moreover, miR-181a dysregulation contributed to memory impairments by modifying Tau protein levels [54]. Similarly, miR-186-5p also directly targeted GluA2 by binding to 3′-UTR of GluA2-coding transcript Gria2 and regulated AMPAR-mediated currents. Overexpression of this miRNA decreased Aβ levels [55,56]. In the PC12 cell AD model, miR-26b, known to be involved in neuronal aging by inhibiting total neurite outgrowth and promoting apoptosis, reduced the expression of its target Neprilysin, an enzyme modulating Aβ concentrations [48]. Targeting the neuritin 3′-UTR, miR-199a decreased the neuritin protein level in APP/PS1 mice, thus accelerating cognitive function impairment [49]. miR-34a was proven to have several roles in regulating Tau expression in vitro (M17D neuroblastoma cell and HEK 293 cell models) [73,74] and synaptic plasticity [57,75]. miR-34a knock out in the APP/PS1 mice ameliorated AMPA and N-methyl-d-aspartate receptor expression [75].

Notably, proinflammatory cytokines, such as tumor necrosis factor alpha (TNF-α), interleukin-6 (IL-6), and Il-10 released by reactive astrocytes and microglia, are involved in AD pathology [29,33]. In the HEK 293T AD cell model and in the in vivo AD rat model, miR-132 (considered a protective agent in AD) inhibited mitogen-activated protein kinase 1 (MAPK) and inducible nitric oxide synthase (iNOS), reduced oxidative stress, and improved cognitive function via the p38 signaling pathway, a member of MAPK family involved in inflammation and apoptosis [60]. By targeting gasdermin D, the executing protein of pyroptosis of glial cells, miR-22 negatively correlated with IL-18, IL-1β, and TNF-α levels in AD patients’ peripheral blood and enhanced the memory ability in APP/PS1 mice [61]. miR-331-3p was the direct target of the von Hippel–Lindau tumor suppressor that has neuroprotective effects. It was downregulated in AD patients’ serum and Aβ40-treated SH-SY5Y cells, and negatively correlated with IL-1β, IL-6, and TNF-α. The overexpression of miR-331-3p enhanced cell viability and inhibited inflammatory responses in Aβ40-treated SH-SY5Y, thus supporting its neuroprotective role [62]. In contrast, miR-485-3p promoted AD severity by targeting AKT3, a gene regulating cell proliferation, apoptosis, and inflammatory response, in Aβ40-treated SH-SY5Y and BV2 cells, positively correlating with the inflammatory response triggered by IL-1β, IL-6, and TNF-α [58].

Autophagy has a neuroprotective role in neurodegenerative diseases, and various miRNAs diversely affect this process [33]. Silencing miR-204 enhanced transient receptor potential mucolipin-1 (TRPML1), the main channel for releasing Ca^2+^ from lysosomes and able to regulate autophagy and Aβ accumulation [63]. miR-204 also promoted reactive oxygen species (ROS) production and inhibited mitochondrial autophagy in AD, activating the signal transducer and activator of transcription 3 (STAT3) pathway in vitro and in vivo [63]. Interestingly, miR-331-3p and miR-9-5p were dysregulated in AD APPswe/PS1dE9 mice. They were downregulated in the early phase of the disease while upregulated in the late one. The overexpression of miR-331-3p and miR-9-5p impaired autophagic activity and promoted Aβ formation [64]. Treating SH-SY5Y cells in vitro with miR-331-3p and miR-9-5p mimics reduced Sequestosome 1, Optineurin, and Beclin1 proteins, while miRNA antagomirs produced the opposite effects on protein expression [64]. These results indicate that miR-331-3p and miR-9-5p regulated Aβ elimination via Sequestosome 1 and Optineurin autophagy receptors [64]. In addition, these miRNA antagomirs ameliorated memory loss and motility decline at a late stage in vivo [64].

Overall, this growing evidence demonstrates the involvement of miRNAs in multiple pathophysiological mechanisms of AD.

### 3.2. The Biomarker Value of miRNAs in Alzheimer’s Disease

Hence, microRNAs have been investigated as biomarkers and therapeutic agents in AD [28,29,55]. Many miRNAs, such as miR-483-5p, miR-29, miR-34, miR-146, miR-125b, miR-501-3p, miR-146a, miR-212, miR-132, miR-107, and miR-132-3p, are dysregulated in the brain or circulating fluids many years before exhibiting AD symptoms [46,73,76,77,78,79]. Furthermore, several studies have been conducted simultaneously on many miRNA-based signatures with advantageous cost, accuracy, sensitivity, and specificity compared to one analysed miRNA [29,80,81,82,83], as outlined in Table 1, which reports the main miRNA suggested as biomarkers in AD.

### 3.3. Therapeutic Implications of miRNA in Alzheimer’s Disease

miRNA-based therapeutic approaches have been broadly evaluated [55,85,86,87]. miR-181a inhibitors decreased soluble and synaptosome-enriched Tau in the hippocampus from 3xTg-AD mice [55]. The administration of miR-124 antagomir attenuated Tau hyperphosphorylation and rescued learning and memory impairments in the P301S mouse model of AD [85]. The treatment with miR-1233-5p, downregulated in Aβ(+)MCI patients’ platelets and megakaryocytes MEG-01 cells, reduced Aβ-increased platelet adhesion to fibronectin and expression of P-selectin [86]. Injecting lentivirus encoding miR-31 into the hippocampus of 3xTg-AD mice reduced Aβ and Vesicular glutamate transporter 1-containing puncta and improved cognitive deficits. In addition, miR-31 overexpression also decreased APP and BACE1 expression in vitro and in vivo [87]. In AD rat models, miR-592 was upregulated and, consequently, its blocking rescued oxidative stress, promoting cell viability by activating the Keap1/Nrf2/ARE antioxidant signaling pathway and upregulating KIAA0319 (targeted gene of miR-592) [88]. miR-204-3p was downregulated in APP/PS1 mice and its overexpression reduced neurotoxicity by inhibiting NADPH oxidase 4, one of its targets, enhanced synaptic and memory functions, and decreased oxidative stress in the hippocampus [89]. In addition, a microRNA-based multitargeted therapeutic was also developed as MG-6267—the dual inhibitor of acetylcholinesterase and miR-15b biogenesis [90]. These data highlight the promising potential of miRNAs in the cure of AD.

## 4. Parkinson’s Disease

Parkinson’s disease (PD) is the second most common neurological disorder after AD, characterized by progressive loss of neurons in the brain, especially dopaminergic (DA) ones, in the substantia nigra pars compacta (SNpc), resulting in cognitive and behavioral dysfunctions [91,92,93,94,95]. The literature reports that 1% of people above 60 years old suffer from PD and approximately nine million individuals worldwide will develop PD by 2030 [91]. The loss of DA neurons and decrease in DA signaling result in motor dysfunction and clinical symptoms such as resting tremor, bradykinesia, rigidity, and postural instability [91]. Besides, the intracellular inclusions of Lewy bodies, enriched with aggregated α-synuclein (α-syn), are also identified in neurons of PD patients, and impair various pathways and activate neuroinflammation [92]. Apart from the SNpc, neuron loss occurs in several other brain regions, such as the amygdala, the vagus nerve’s dorsal motor nucleus, the hypothalamus, cortex, and thalamus [93]. First motor dysfunctions develop after about a 70% loss of DA neurons in the SNpc. The preclinical phase is estimated to last 8–17 years, indicating the existence of complex mechanisms in the early PD phases [96]. Therefore, the availability of preclinical PD biomarkers is essential to design future neuroprotective strategies for high-risk patients.

### 4.1. miRNA Pathological Traits in Parkinson’s Disease

Several specific miRNAs have been implicated in PD pathogenesis and they are involved in the following molecular mechanisms:

(i) Autophagy (downregulated: miR-181b [97]; upregulated: miR-3473b [98]);
(ii) Neuronal survival (upregulated: miR-421 [99]);
(iii) Mitochondrial function (downregulated: miR-5701 [100]);
(iv) Pyroptosis (downregulated: miR-135b [101]);
(v) α-syn regulation (downregulated: miR-26a, miR-425 [102,103], and miR-30 [104]);
(vi) Neurotoxicity and inflammation (upregulated: miR-9-5p [105], miR-494-3p [106], miR-543-3p [107], and miR-421 [99]; downregulated: miR-29c-3p [108]).

Several studies have demonstrated the aberrant expression of many miRNAs in in vitro [97,99,100,101] and in vivo PD mouse models [102,103,105,106,107]. miR-421, known to regulate myocyte enhancer factor 2D (a DA neuron survival modulator) expression negatively, was found to increase in in vitro and in vivo PD models [99]. miR-181b was decreased in the 1-methyl-4- phenylpyridinium ion (MPP^+^)-treated PC12 cell model of PD [97]. In this in vitro model, overexpression of miR-181b inhibited autophagy and increased cell viability via targeting the PTEN/Akt/mTOR signaling pathway [97]. miR-5701 was downregulated in 6-hydroxy dopamine-treated SH-SY5Y cells, another model of PD, and it negatively regulated Valosin-containing proteins (VCP) that are involved in lysosomal degradation pathways [100]. Moreover, by targeting VCP, miR-5701 regulated mitochondrial function by increasing mitochondrial DNA and decreasing mitochondrial complex I activity and adenosine triphosphate (ATP) formation [100]. MiR-135b, known to target *FoxO1* by negative feedback, was downregulated in MPP^+^ PD modelled SH-SY5Y and PC-12 PD cells [101]. Accordingly, miR-135b mimics attenuated the toxic effects of MPP^+^ in vitro on pyroptosis, downregulating NLR family pyrin domain containing 3 (NLRP3) and Caspase-1 [101]. In a PD mouse model, miR-26a, which represses the death-associated protein kinase 1 (DAPK1), increased in PD mice, was downregulated [102]. The downregulation of miR-26a and upregulation of DAPK1 induced cytotoxic increase in α-syn that caused DA neuron death in vivo [102]. In the in vivo 1-methyl-4-phenyl-1,2,3,6-tetrahydropyridine (MPTP)-treated PD mouse model, the miR-425 level, which correlates to receptor-interacting protein kinase 1 expression, was downregulated [103]. miR-9-5p, which directly targets STAT1, was shown upregulated in MPP^+^-treated SH-SY5Y cells, thus developing a neurotoxic phenotype [105]. miR-494-3p caused neurotoxicity in two PD cell models via regulating brain-derived neurotrophic factor (BDNF) levels [106], and miR-543-3p reduced glutamate transporter type 1 expression both in in vitro and in vivo models [107]. Other related miRNAs involved in PD, such as miR-29c-3p, miR-30, and miR-3473b, are reported below in the therapeutic section [98,104,108].

Several studies demonstrated the dysregulation of different miRNAs also in PD patients, such as downregulation of miR-150 in serum [109], hsa-miR-626 in CSF [110], miR-218, miR-124, and miR-144 in prefrontal cortex brain samples [111], and miR-425 in the postmortem midbrain [103]. Similarly, several upregulated miRNAs have been identified [105,106,107,112,113,114]: miR-27b-3p in blood [112], miR-153, miR-409-3p, and miR-10a-5p in CSF extracellular vesicles (EVs) [113], miR-21-3p, miR-224, miR-373-3p, miR-26b, miR-106a, and miR-301b in SNpc [114].

### 4.2. The Biomarker Value of miRNAs in Parkinson’s Disease

The diagnostic criteria for PD are based on clinical signs of motor functions, but the main issue is that PD can only be diagnosed once the DA neuron loss reaches up to 70% [115]. Therefore, the need for molecular biomarkers as potential clinical tools to diagnose PD is obvious. The biomarkers for PD could be the PD-related proteins in the CSF and brain tissues, such as α-syn for protein aggregation and Lewy body formation or protein Deglycase 1 (DJ-1) for mitochondrial dysfunction [116]. Blood and plasma samples are the ideal biomarker source, and miRNAs obtained from plasma are more abundant, tissue-specific, and stable. Circulating miRNAs can be used as noninvasive biomarkers, promoting the early PD detection and controlling the progression of the pathology [117]. Table 2 presents some miRNAs recently proposed as promising PD biomarkers.

### 4.3. Therapeutic Implications of miRNA in Parkinson’s Disease

Treatments for PD include several approved medications (Levodopa, dopamine receptor agonists, catechol-O-methyl transferase inhibitors, and monoamine oxidase B inhibitors) [95], but there is also a variety of potentially effective compounds of natural origin under investigation (e.g., Mucuna pruriens [122]; ursolic acid [123]; chlorogenic acid [124]). More recently, different miRNA-based approaches are being investigated to cure PD. miRNA mimics and anti-miRNAs may represent useful tools to re-establish the physiological level of miRNAs in PD models, thus being promising as novel therapeutic tools. miR-150 levels in serums of PD patients were downregulated compared to healthy controls (HC) and its concentration negatively correlated with the proinflammatory cytokine levels (IL-1β, IL-6, and TNF-α) [109]. The restoration of miR-150 by mimics in lipopolysaccharide (LPS)-treated BV2 cells reduced the above-reported inflammatory cytokines via targeting the *AKT3* gene [109]. miR-29c-3p mimics inhibited microglia activation and suppressed NLRP3 inflammasome in in vitro PD mouse models through directly targeting the nuclear factor of activated T cells 5 (NFAT5) [108], and miR-135b mimics attenuated pyroptosis [101]. The injection of AAV2 or AAV8-miR-30 human α-syn mimics into the SN rescued TH-positive dopamine neuron loss and reduced the forelimb deficits in PD rat models [104]. On the other side, the injection of antagomiR-421 into SNpc protected DA neurons in 6-OHDA-treated PD mice [99]. The intracerebral administration of agomiR-425 into SNpc reduced MPTP-induced necroptosis, restored locomotor impairments, and increased dopamine levels in the striatum in a PD mouse model [103]. The treatment with lentivirus-containing antisense miR-543-3p into SN locally and unilaterally in PD mice reduced the DA neuronal injury and α-syn aggregation levels, increased TH-positive cell numbers, and improved motor performance [107]. The injection of miR-3473b antagomir into the midbrain of PD mice enhanced autophagy and inhibited microglia activation via targeting TREM2/ULK1 [98]. Moreover, its inhibition also attenuated LPS-induced BV2 microglial activation [98]. These results are promising for a future potential therapeutic approach in PD treatment.

## 5. Multiple Sclerosis

Multiple sclerosis (MS) is a progressive autoimmune CNS disease characterized by inflammatory demyelination. It is the leading cause of nontraumatic neurological disability in young adults, and it is more common in women than men [125]. The most affected areas of the CNS are periventricular white matter, optic nerve, spinal cord, brain stem, and cerebellum. The main clinical symptoms include muscle weakness, blurred vision, dizziness, fatigue, and gate problems [126]. Several factors are responsible for the pathogenesis of MS and include genetic, epigenetic, microbial, and environmental causes [127]. Therefore, the aetiology and mechanisms of the disease are still not clear. Furthermore, there is no cure for this disease, although there are several effective disease-modifying treatments [128]. Current research on the pathophysiological changes occurring in MS reports an increase in proinflammatory miRNAs and related pathogenic biomarkers, pointing out that there is a great need for MS treatment as well as for understanding the mechanisms of disease progression [129].

### 5.1. miRNA Pathological Traits in Multiple Sclerosis

Several specific miRNAs have been implicated in MS pathogenesis and they are involved in the following molecular mechanisms:

(i) Cell differentiation (downregulated: miR-124 [130]);
(ii) Microglial activation and inflammation (downregulated: miR-155 [131], 467b [132], and miR-146a [133]; upregulated: miR-873 [134];
(iii) Oligodendrocyte differentiation and myelin formation (downregulated: miR-219 [135]; upregulated: miR-17-5p [136] and miR-125a-3p [137]);
(iv) Fibrosis (downregulated: miR-219-5p [138]);
(v) Autophagy (mixed regulation: miR-223 [139,140].

The primary glial cells such as microglia, oligodendrocytes, and astrocytes are abundant in the CNS. They are involved in inflammatory reactions and signal transmission and provide nutritional support to the neuronal cells. They also help in cellular regeneration and repair [127]. A study showed that the expression of miR-124 was significantly lower in activated microglia in the experimental autoimmune encephalomyelitis (EAE) mouse model [129]. miR-124 negatively regulated the CCAAT/enhancer-binding protein α (CEBPα) involved in myeloid cell differentiation [130]. Furthermore, in vivo administration of miR-124 reduced lymphocytes, CD4^+^ T cells, and macrophages, and activated CD45^hi^ microglia [130]. Another study evaluated the role of miR-155 in macrophages and microglia activation by transfecting cells with miR-155 analogs/mimetics. The authors demonstrated that miR-155 analogs/mimetics significantly increased reactive microglia and the secretion of inflammatory factors [131]. miR-219, which is deficient in MS, plays a fundamental role in the regulation of oligodendrocyte differentiation and myelin formation [135]. miR-219 enhanced the myelination process in aging rats when delivered intranasally through serum-derived exosomes [135]. miR-17-5p was upregulated in CD4^+^ lymphocytes isolated from MS patients [136]. In this in vitro model, an antimiR-17 upregulated phosphatidylinositol 3-kinase (PI3K) regulatory subunit 1, a tumor suppressor, and PTEN, a PI3K inhibitor [136]. A study reported that miR-125a-3p was upregulated in MS patients and oligodendrocyte precursor cells (OPC) isolated from the spinal cord of EAE mice [137]. Overexpression of miR-125a-3p by lentiviral-operated administration into the subcortical white matter of the lysophosphatidylcholine-induced demyelination MS model, resulted in impairing OPC maturation and inhibiting remyelination [137]. miR-873, which promotes NF-κB activation and increases inflammatory factors such as IL-6, macrophage inflammatory protein-1, and monocyte chemotactic protein -2, was upregulated in astrocytes from EAE mice [134]. Other dysregulated miRNAs, such as the downregulated miRNA-467b, miR-146a, and miR-219-5p [132,133,138,139,141,142,143,144,145,146,147,148,149,150,151,152,153,154,155,156,157,158], or the altered regulation of miR-223 were proposed to participate in MS pathology and have potential therapeutic application [132,138,139,140,150].

### 5.2. The Biomarker Value of miRNAs in Multiple Sclerosis

Several studies published in recent years have demonstrated that miRNAs work as important diagnostic biomarkers of MS (Table 3). The plasma samples were collected from both MS patients and healthy donors to perform miRNA gene chip analysis, and the results showed the upregulation of miR-22, miR-422-a, miR-572, miR-614, miR-648, and miR-1826, and the downregulation of miR-1979 [153]. Similarly, miR-145 was overexpressed in PBMCs of MS patients [154]. Another study showed the overexpression of miR-145 in peripheral blood, thus being used as a biomarker in MS patients [155]. Moreover, MS patients can also be diagnosed by evaluating the overexpression of miR-320a, miR-572, miR-27a-3p, and miR-199a-5p in serum [156]. On the other hand, MS patients showed downregulation of miR-572 in serum compared with HC [157]. Some miRNAs also correlate with different phases of the disease, such as miR-326 and miR-26a, that can distinguish between the relapsing and remitting phases of MS [158]. A study identified nine miRNAs (miR-15b-5p, miR-23a-3p, miR-30b-5p, miR-223-3p, miR-374a-5p, miR-342-3p, miR-432-5p, miR-433-3p, and miR-485-3p) that could discriminate relapsing–remitting from progressive MS [141].

### 5.3. Therapeutic Implications of miRNA in Multiple Sclerosis

RNA interference technology plays an important role in regulating miRNA content in MS [132]. The injection of miRNA-467b mimics in mouse-spleen-derived CD4^+^ T cells led to the downregulation of Th17 differentiation by targeting eukaryotic initiation factor 4 F (*eIF4E*), preventing infiltration of inflammatory cells into CNS, and delaying disease progression in the EAE mouse model of the disease [132]. Moreover, a neutral lipid emulsion containing miR-146a mimics were shown to cross the blood–brain barrier (BBB), increasing the M2 microglia/macrophage phenotype, rescuing OPC differentiation, enhancing remyelination, and improving the neurological in vivo outcomes via negatively affecting toll-like receptor 2/interleukin-1 receptor-associated kinase 1 signaling pathway [150]. miR-223 directly targets the autophagy related 16-like 1 (Atg16l1) and its deficiency augmented autophagy in the EAE mouse brain microglial cells. Overexpression of miR-223 decreased the cellular level of Atg16l1 in the LPS-induced autophagy model in BV2 cells [139]. In EAE mice, the administration of miR-219-5p through the tail vein negatively regulated fibronectin 1 expression, blocked bladder fibrosis, and controlled smooth bladder muscle tone [138]. In contrast, antagomiR-125a-3p stimulated oligodendrocyte maturation in vitro since miR-125a-3p targets Neuregulin1, Tyrosine kinase protein Fyn, the small GTPase Ras homolog family member A (RhoA), and p38, regulating myelin basic protein mainly expressed in mature/myelinating oligodendrocytes [151]. Obstacles to the miRNA-based therapeutic approach in ND in general and MS are the off-target effects due to multiple target genes and difficulty in crossing the BBB. Therefore, the development of novel delivering methods, such as nanosystems, biomaterials, EVs, gene therapy (lentivirus vectors), and stem cell implants, deserves to be investigated [152].

## 6. Huntington’s Disease

Huntington’s disease (HD) is a neurodegenerative disease caused by CAG repeat expansion in the Huntingtin gene (*HTT*), including a complex net of pathogenic mechanisms [159,160,161]. HD is the most common of the nine polyglutamine diseases [162], with a prevalence of ~12 per 100,000 individuals in European populations [163]. The motor onset occurs from childhood to old age, with a mean age around 45 years [164]. Currently, there is no effective treatment, and patients usually die 10–20 years after illness onset [165]. HD symptoms include progressive involuntary choreiform movements, behavioral and psychiatric disturbances, and dementia [161]. Recently, miRNA-expression dysregulation has been reported in many studies using different HD human samples [166,167,168,169] and animal models [170,171,172,173].

### 6.1. miRNA Pathological Traits in Huntington’s Disease

Several specific miRNAs have been implicated in HD pathogenesis and they are involved in the following molecular mechanisms:

(i) Neuronal development and survival (downregulated: miR-212, miR-128, miR-218, miR-124, and miR-132 [171,174]);
(ii) Neuronal differentiation and morphology (downregulated: miR-124 [170] and miR-196a [175]);
(iii) mHTT aggregation (downregulated: miR-128a [172], miR-181c, and miR-133 [176]; upregulated: miR-194 [176]);
(iv) Synaptic function (upregulated: miR-140 [166]);
(v) Cell apoptosis (downregulated: miR-34a [84]).

In a study including 15 HD patients and seven controls, the isolated miRNAs from plasma samples were analysed and 168 dysregulated miRNAs were found in symptomatic patients, namely: miR-877-5p, miR-223-3p, miR-223-5p, miR-30d-5p, miR-128, miR-22-5p, miR-222-3p, miR-338-3p, miR-130b-3p, miR-425-5p, miR-628-3p, miR-361-5p, and miR-942 were significantly increased, while miR-122-5p, miR-641, and miR-330-3p levels were decreased compared with controls [168]. In the PREDICT-HD study, miRNA levels were measured in CSF using the HTG protocol, and six miRNAs (miR-520f-3p, miR-135b-3p, miR-4317, miR-3928-5p, miR-8082, and miR-140-5p) were significantly increased in the prodromal HD-gene-expansion carriers versus controls [169].

In animal models, CAG length-dependent microRNA expression was altered in the mouse brain. In particular, 159 microRNAs were altered in the striatum, 102 in the cerebellum, 51 in the hippocampus, and 45 in the cortex [170]. Among them, miR-212, miR-132, miR-218, and miR-128, associated with aspects of neuronal development and survival, were found to be downregulated [171]. In a monkey model, miR-194 level was upregulated, whereas miR-181c, miR-128, and miR-133 expressions were downregulated in the frontal cortex region [172]. In addition, this study also confirmed HD-signaling genes regulated by miR-128a, including HTT and Huntingtin interaction protein 1, have a crucial role in the disease.

Some dysregulated miRNAs, such as miR-140-5p, miR-124, and miR-34a-5p, contribute to the HD pathology. miR-140 is a negative regulator of disintegrin and metalloproteinase 10 (ADAM10) [169]—that is increased in HD—accumulating at the postsynaptic densities and causing excessive cleavage of the synaptic protein N-cadherin, which produces a detrimental role at the HD synapses [177]. miR-124 is one of the crucial regulators for neuronal differentiation in neurodegeneration [170] and there was a decrease in *STHdh^Q111^/Hdh^Q111^* HD cells and mice models [178]. The expressions of the miR-34 family members were investigated in the brain, liver, and skeletal muscle from R6/2 mice, and the results demonstrated that miR-34a-5p was more expressed than miR-34-b/c isoforms in all three tissues [173]. This study also proved age- and genotype-dependent downregulation of miR-34a-5p in the brain. miR-34a also positively interacted with cell cycle progression, cellular senescence, and apoptosis [84]. Other roles of miRNAs present in the literature are reported in Table 4.

### 6.2. The Biomarker Value of miRNAs in Huntington’s Disease

There is currently an urgent need for biomarker measure methods consistent with HD pathology, and the development of miRNA biomarker assays may contribute as a significant indicator for HD progression diagnostic [166]. Some studies focused on detecting specific miRNA [166,167]; others figured out several miRNA-signature alterations [161,168,169].

miR-9* was downregulated in peripheral leukocytes of HD patients and supposed to increase the expression of the corepressor of repressor element 1-silencing transcription factor [166]. miR-34b was elevated in mHTT-expressing NT2-derived neurons and in plasma samples of HD patients [167]. Moreover, the elevated expression of miR-34b appeared prior to symptom onset that was affordable for early detection of HD, needing a sample volume as small as 10 µL [167]. The circulating miRNAs from plasma or CSF samples were investigated to explore miRNA signatures [168,169]. Table 5 reports miRNAs as potential biomarkers in HD.

However, the general limitations of these studies are the sample size, the unknown interactions of extrinsic factors, such as nutrition, medications, ethnicity, or race, as well as technical issues, such as accurate detection methods or internal reference for miRNA expression [161,166,167,168,169]. Therefore, additional analysis of larger cohorts during disease progression will undoubtedly improve the efficacy of these measures.

### 6.3. Therapeutic Implications of miRNA in Huntington’s Disease

Currently, miRNA-based therapeutics are being developed to target mutant-HTT [170,175,179,180,181]. By injecting miRNA-124 in mice, the two neuroprotective molecules peroxisome proliferator-activated receptor-coactivator-1 alpha (PGC-1α) and BDNF were increased, while the SRY-related HMG box transcription factor 9, a repressor of cell differentiation, was downregulated [170]. The role of miR-196a was examined in cultured primary cortical neurons isolated from FVB mouse embryos and miR-196a-overexpressing transgenic mice [175]. The results showed that miR-196a improved neuronal morphology by suppressing the expression of RAN-binding protein 10 and increasing β-tubulin polymerization, and ameliorated intracellular transport, synaptic plasticity, learning, and memory abilities [175].

Despite the improved knowledge about miRNA alterations in HD, only some studies on miRNA-based therapeutic delivering strategies have been conducted in different in vivo models. An exosome-based delivery method was developed to inject miRNA-124 into the striatum of R6/2 transgenic HD mice, and it reduced the target protein RE1-silencing transcription factor, a regulator of the neurogenesis [179]. However, in that study, the behavioral performances were not improved due to the critical issues of the delivery method. Recently, many other studies have shown that artificial miRNAs can reduce mutant HTT in small and large HTT animal models [180,181]. An AAV5-encoded miRNA targeting human HTT was recently administrated into the striatal region of the Hu128/21 mouse model to lower the different HTT isoform expression [180]. The outcomes of that study showed a behavioral improvement and a long-lasting reduction of wild-type HTT [180]. Pfister and Coll. (2018) also applied a single administration of scAAV9-miRHTT into HD sheep striatum and recorded a reduction of the human mutant HTT mRNA in caudate and putamen at 1 and 6 months postinjection [181]. We can conclude that miRNA-mediated gene therapy is promising in treating of HD.

## 7. Amyotrophic Lateral Sclerosis

Amyotrophic lateral sclerosis (ALS) is the most frequent motor neuron (MN) disease that affects motor neurons in the motor cortex, brainstem, and spinal cord [182,183]. Approximately 90% of ALS cases are sporadic (sALS), while 10% are familial (fALS), defined by the occurrence of ALS in more than one family member [145]. Around 30 different genes and more than 100 mutations are linked to ALS. The most frequent gene mutations are chromosome 9 open reading frame 72 (*C9orf72*), Superoxide dismutase type 1 (*SOD1*), TAR DNA-binding (*TARDBP*), and fused in sarcoma (*FUS*) [184,185,186,187]. The pathophysiological mechanisms of MN degeneration remain largely unknown. ALS is a complex disease in which multiple cell types, such as astrocytes, microglia, oligodendrocytes, Schwann cells, and skeletal muscle cells, have important roles in the pathology [188,189]. Different cellular and molecular mechanisms contributing to ALS include protein misfolding and aggregation, mitochondrial dysfunction, neuroinflammation, oxidative stress, axonal transport deficits, glutamate excitotoxicity, RNA dysfunction, neuromuscular junction abnormalities, cytoskeletal derangements, dysregulation of growth factors, and abnormal calcium metabolism [188]. In this context, several studies have investigated the dysregulation of miRNAs, thus pointing out that the miRNA signature could be a valuable tool to identify ALS biomarkers and therapeutic targets [190].

### 7.1. miRNA Pathological Traits in Amyotrophic Lateral Sclerosis

Several specific miRNAs have been implicated in ALS pathogenesis and they are involved in the following molecular mechanisms:

(i) Autophagy (downregulated: miR-335-5p [191]);
(ii) Apoptosis (downregulated: miR-183-5p [192]);
(iii) MN excitability (downregulated: miR-218 [193]);
(iv) Neuronal differentiation and neuromuscular junction (upregulated: miR-129-5p [194]);
(v) Neuroinflammation (upregulated: miR-142-3p [195]).

Multiple miRNAs are imbalanced in ALS, corrupting synapses/neuromuscular junction function, neurofilaments, neurogenesis, and RNA/protein metabolism [190,191,192,193,194,195,196]. For instance, miR-335-5p, miR-183-5p, and miR-218 expression is downregulated in ALS patient serum, ALS cells, or mouse models, thus affecting several disease-linked mechanisms [191,192,193]. miR-335-5p is decreased in ALS patient serum and directly targets caspase-7 in SH-SY5Y neuronal cells [191]. After 72 h, SH-SY5Y cells transfected with an miR-335-5p inhibitor showed abnormal autophagy processes and activated caspase 3/7 apoptotic pathways [191]. miR-183-5p was downregulated in ALS patients and in the spinal cord of SOD1^G93A^ mice at the late symptomatic stage of the disease. However, miR-183-5p was upregulated in the early phase [192]. When transfected in NSC-34 cells, miR-183-5p mimics protected cells from death, while inhibitors induced cell death under stress conditions [192]. Using sequence analysis, the authors reported that miR-183-5p affected apoptosis and necroptosis in NSC-34 cells by targeting the receptor-interacting serine/threonine-protein kinase 1, a necroptosis regulator, and programmed cell death 4, a critical protein in cell apoptosis [192]. After confirming the miR-218 downregulation in human spinal MNs, it emerged that miR-218 genetic variants target the potassium channel Kv10.1, disrupting in vitro the excitability of primary rat MN cultures [193].

On the other side, other miRNAs are upregulated in ALS, including miR-129-5p, miR-5572, and miR-142-3p [194,195,196]. Although limited knowledge is available regarding miR-129-5p, it seems to maintain the neuronal function and homeostasis and regulate neuronal differentiation, possibly targeting the RNA-binding protein ELAVL4/HuD [194]. Moreover, miR-129-5p was dysregulated in different ALS disease paradigms both in vivo in SOD1^G93A^ mice and sALS patients, and in vitro. In vivo silencing miR-129-5p increased the lifespan of SOD1^G93A^ mice and rescued the neuromuscular junction degeneration [194]. Overexpression of miR-129-5p in NSC-34, SOD1^G93A^, and SH-SY5Y/SOD1^G93A^ cells decreased HuD level, a crucial protein for neuronal development and maturation [194]. miR-129-5p also inhibited neurite outgrowth in SH-SY5Y/SOD1^G93A^ cells [194]. miR-5572 is a recently discovered molecule in humans, and its function is still unclear [194]. Moreover, miR-5572 binds the 3′-UTR of the targeted *SLC30A3* gene and is increased in the spinal cord of sALS patients [196]. miR-142-3p was altered in some NDs, such as AD or MS, and non-NDs, such as diabetes or heart failure. In ALS, miR-142-3p is associated with neuroinflammation and microglial activation and was predicted to target both *TDP-43* and *C9orf72* genes. Moreover, it increased in serum of the SOD1^G86R^ and TDP43^A315T^ mouse models of the disease and sALS patients [195]. A clinical study in *C9orf72* patients demonstrated miR-34a-5p and miR-345-5p overexpression, while miR-200c-3p and miR-10a-3p were downregulated in correlation with the disease stage [197]. Table 6 summarizes the miRNAs altered in ALS patients.

### 7.2. The Biomarker Value of miRNAs in Amyotrophic Lateral Sclerosis

miRNAs are secreted in the CSF and their analysis in this fluid could be used for clinical diagnosis. In addition, miRNAs are also muscle-specific and, therefore, they may have a broad application as biomarkers in ALS [190,202]. In a cohort of 20 ALS/motor neuron disease patients and 20 controls, some miRNAs were isolated from a neural-enriched subpopulation of EVs from total plasma samples and confirmed eight miRNAs differently expressed with respect to controls. In detail, miR-146a-5p, miR-199a-3p, miR-151a-3p, miR-151a-5p, and miR-199a-5p were upregulated in ALS patients, while miR-4454, miR-10b-5p, miR-29b-3p, and miR-151a-5p were downregulated [203]. In a study including 14 ALS patients, 9 nonALS neurological disease controls, and 9 healthy controls, CSF samples showed evidence of a positive correlation between EV-derived miR-124 levels and the disease severity (indicated by ALSFRS-R score) of male patients [204]. Another study collected muscle biopsy samples from 19 ALS patients to validate miRNAs and showed that only miR-206 levels negatively correlated with the muscle strength, assessed using a medical research council grading scale [205]. However, due to the limitation of the sample size, biological sources, and mixed hereditary causes, further studies are needed before using these miRNAs for clinical diagnosis [206].

### 7.3. Therapeutic Implications of miRNA in Amyotrophic Lateral Sclerosis

The pharmacological treatment based on miRNAs as a novel therapeutic approach in ALS has been exploited in numerous preclinical studies by stimulating or inhibiting miRNA production via different delivering techniques, such as adeno-associated virus vectors (AAV), EVs, or antisense oligonucleotides [202]. miR-494-3p secreted in EVs from inducible neural pluripotent cell-derived astrocytes was downregulated in astrocytes prepared from patients carrying the *C9orf72* mutation and healthy controls. Treating HB9-GFP^+^ mouse MNs with an miR-494-3p mimic rescued the neurite length and number of nodes per cell and increased MN survival [206].

Two miRNAs, miR-101 and miR-451, delivered by AVV5 and targeting *C9orf72* to silence its expression, reduced the *C9orf72* mRNA expression in both the nucleus and cytoplasm in two ALS cell models, namely HEK293T and induced pluripotent stem cell (iPSC)-derived frontal brainlike neurons from a patient affected by frontotemporal dementia (FTD). They also inhibited the formation of nuclear RNA foci in (G_4_C_2_)_44_-expressing HEK293T cells [207]. These data would support the feasibility of miRNA-based and AAV-delivered gene therapy to reduce the gain of toxicity in ALS and FTD patients.

The dysregulation of the hsa-miR-17~92 cluster/nuclear PTEN pathway was evidenced in SOD1^G93A^ mice before the disease onset. Overexpressing miR-17~92 via self-complementary AAV9 delivering prolonged the survival of SOD1^G93A^ mice and ameliorated the neuromuscular function; besides, the hsa-miR-17~92 deletion provoked severe loss of MNs in the lateral motor column in the spinal cord. Finally, the survival of human iPSC-derived SOD1^+/L144F^ MNs was extended [208]. Therefore, miR-17~92 may be valuable as a prognostic marker of MN degeneration and a therapeutic target in SOD1-linked ALS. On the other hand, genetic ablation of one or two miR-155 alleles in SOD1^G93A^ mice reduced the expression of the proinflammatory genes *Tnf*, *Fasl*, *Ccl2*, and *Nos2* in the spinal cord microglia and *Tnf*, *Il1b*, *Fasl*, *Nos2*, and *CCR2* in Ly6C^Hi^ splenic monocytes. Partial or total miR-155 deletion reversed the expression of abnormal proteins in the spinal cord and preserved the phagocytic function of microglia in vivo. Moreover, antimiR-155 administration to SOD1^G93A^ mice increased rotarod performance, delayed disease onset, and extended survival [209]. In SOD1^G93A^ mice, miR-29a-antagomirs, administered in vivo ICV, maintained muscular strength longer than vehicle-treated mice and tended to improve lifespan [210]. The available evidence suggests that miRNAs may represent a promising tool for ALS treatment. However, further studies are needed to evaluate efficacy and safety, figure out effective delivering methods, deepen knowledge of the molecular pathways related to disease, and verify the results in patients.

## 8. miRNA Engagement Overlapping in Neurodegenerative Diseases

**Figure 2:**
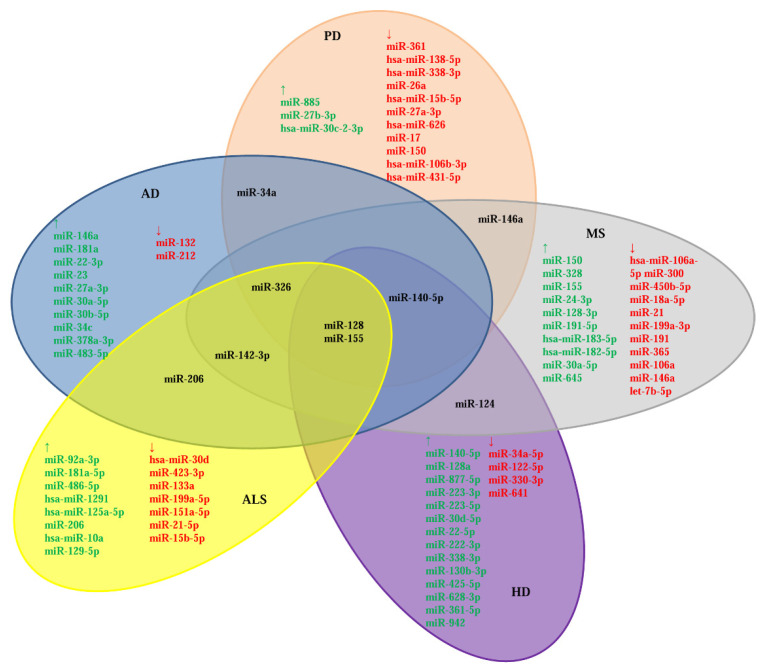
miRNA interconnection among NDs. ↑: upregulated; ↓: downregulated.

Several studies have identified some miRNA dysregulation in one specific ND, whereas others have focused on the influence of one miRNA in different NDs. However, the miRNAs’ role across several NDs still needs further study. Figure 2 represents specific miRNAs shared among NDs.

Recently, a PRISMA-based review reported that miR-146a-5p, miR-155-5p, and miR-223-3p were upregulated in tissues and animal models of 12 NDs including AD, HD, ALS, PD, and MS; meanwhile, miR-9-5p, miR-21-5p, the miR-29 family, miR-124-3p, and miR-132-3p exhibited mixed regulation [211]. Here, we summarized the literature on the diverse dysregulation of miR-128, miR-140-5p, miR-206, miR-326, and miR-155 (Table 7).

As mentioned above, there was a diversified regulation of miR-128 levels related to the oxidative stress mechanism in AD, PD, and HD [212], involving the TrkC.T1 receptor and the TNF-α level in astrocytes in ALS [213], and regulation of pleiotropic cytokine TGFβ related to T-helper 17 (Th17) cells in immunological effects in MS [214].

The miR-140-5p involvement in AD included mitochondrial dysfunction, autophagy, Aβ, and Tau accumulation and free radical production [180,213], whereas miR-140-5p dysregulation in HD was involved in excitatory synapse function, in increased postsynaptic proteolysis, and in electrophysiological alterations due to ADAM10 hyperactivity [177]. In PD, miR-140-5p induced inflammation via the TRL4/NFκB signaling pathway [215], while in MS it inhibits Th17 differentiation by interacting with OIP5-AS1 and RhoA/ROCK2 signaling [216] or Th1 differentiation via DNA methylation and mitochondrial respiratory pathway [217].

miR-206 enhanced the detrimental effects of Aβ42 by suppressing the expression of BDNF in AD [218,219,220]. In the ALS SOD1^G93A^ mouse model, the concentration of miR-206, which is supposed to participate in neuromuscular junction (NMJ) activity, gradually increased with age in muscle biopsy samples [205]. miR-206 suppresses the Histone deacetylase 4 (HDAC4), which mediates the nerve–skeletal muscle interaction factor in muscle isolated from miR-206^−/−^ mice. miR-206 also mediated fibroblast growth factor binding protein 1, a factor promoting NMJ regeneration. Its expression decreased in the miR-206^−/−^ mouse model [205]. The cellular mechanisms related to the influence of miR-155 in AD, HD, ALS, PD, and MS in general included BBB permeability, apoptosis, neurite outgrowth, and microglia activation [59,131,181,221,222,223,224,225,226,227]. miR-326 decreased Aβ and Tau tangle formation, attenuated apoptosis, improved cell viability, and downregulated stress proteins in AD [228,229]; sustained axon development and regulated neuron death via BDNF1 and HIF1 in ALS [230]; inhibited iNOS activation and suppressed DA neuron apoptosis in PD [231,232]; and induced Th17 differentiation and maturation in MS [233].

miRNAs’ administration to reach intracellular space may occur through different delivering methods, such as viral vectors, nonviral tools, liposomes, nanoparticles, or EVs [10]. miR-155 was delivered by AAV5 or AAV9 vector in HD in vivo models [181,223]. miR-155 was upregulated in PD mouse produced by AAV2-α-syn injection, and deletion of miR-155 in miR-155^−/−^ mice reduced the proinflammatory action of α-syn in primary microglia [225]. Hence, exposing microglia from miR-155^−/−^ mice to a synthetic mimic miR-155 reversed this effect [225]. In ALS SOD1^G93A^ mice, an antimiR-155 was delivered via an osmotic pump directed into the lateral ventricles [11]. miR-326 was delivered via a lentivirus vector in an AD mouse model [228] or EVs derived from T-cells in RRMS patients [206,233]. Typically, the viral delivery had high efficiency and prolonged suppression of miRNA, while the remaining delivery methods were less toxic and characterized by fewer limitations of the DNA size [11]. Therefore, developing an appropriate delivering method of miRNAs to targets is necessary to improve the efficacy of the treatment.

Table 7 summarizes the involvement of miRNAs in different NDs, reporting the targets, the up- or downregulation, and the model source.

**Table 7:** Summary of miRNA dysregulation across NDs.

miRNA	ND	Target	MiRNA Expression and Model	Reference
miR-128	AD	*STIM2*	↑ Male *APP/PS1* mice	[234]
		*ARPP21*	↑ In vitro, NMRI mice	[235]
		Not mentioned	↑ human plasma, CSF of AD patients	[29]
	HD	Not mentioned	↓ YAC128 and R6/2 mice	[174]
		mHTT	↓ frontal cortex of HD monkey model	[172]
		Not mentioned	↑ Human plasma of HD patients	[168]
			↓ Human HD post-mortem brain	[167]
	ALS	*ABCG1, LGALS3, CTDSP1, BAX*	↓ Blood samples from sALS patients	[236]
		*TrkC.T1*	↓ SOD1^G93A^ mice, post-mortem sALS patient spinal cord	[213]
	PD	*AXIN1*	↓ In vitro, PD mice	[237]
	MS	*BMI1*	↑ T cells isolated from MS human blood	[214]
miR-140-5p	AD	*ADAM10*	↑ post-mortem human AD hippocampus, in vitro	[238]
		*PINK1*	↑ AD rats, in vitro	[239]
	HD	Not mentioned	↑ CSF sample from HD human	[169]
		*ADAM10*	↑ R6/2 and zQ175 mice, postmortem HD patient brain	[177]
	PD	*TLR4*	↓ Blood, colon tissues from PD patients; PC12 cell model	[215]
	MS	RhoA/ROCK2	↓ In vitro, EAE mice, blood MS patients	[216]
		*STAT1* and *Tbx*	↓ Splenic CD4^+^T cells isolated from EAE mice	[217]
miR-155	AD	Microglia fibrillar Aβ_1-42_	↑ In vitro	[221]
		IL-1β, IL-6, TNF-α, Capase-3	↑ hippocampus of AD rats	[59]
		APP	↑ *APP/PSEN1* mice, AD human post-mortem brain	[222]
	HD	mHTT	↓ AAV5 vector, HD rats	[223]
		mHTT	↓ AAV9 vector, HD sheeps	[181]
	ALS	C/EBPβ, Smad2, MFG-E8	↑ SOD1^G93A^ mice	[224]
	PD	α-synuclein	↑ AAV2-SYN mice	[225]
		Not mentioned	↑ Blood of PD patients	[226]
	MS	*SOCS1*	↑ Blood monocytes, myeloid cells from brain lesion in RRMS patients	[131]
		Pro-inflammatory cytokines, myelination/microlia	↑ Brains of MS-cuprizone-induced mice	[227]
miR-206	AD	*BDNF, SIRT1*	↑ Serum of AD patients	[218]
		*BDNF*	↑ Brain of AD patients	[219]
		*BDNF*	↑ APP/PS1 transgenic mice	[220]
	ALS	*HDAC4*	↑ SOD1^G93A^ mice	[205]
		Not mentioned	↑ Blood of sALS patients	[240]
miR-326	AD	*VAV1*	↓ APPswe/PS1d E9 double transgenic mice	[228]
		*PKM2, lncRNA RPPH1*	↓ In vitro	[229]
	ALS	*BDFN1, HIF-1*	↑ Blood and neuromuscular junction of sALS patients	[230]
	PD	*KLK7* gene/MAPK signaling	↓ PD mice	[231]
		*XBP1* gene/JNK signaling	↓ PD mice	[232]
	MS	Th17	↑ T Cell-derived EVs of RRMS patients	[233]

AAV: adeno-associated viruses; Aβ: amyloid beta; APP: amyloid-beta precursor protein; ALS: amyotrophic lateral sclerosis; AD: Alzheimer’s disease; C/EBPβ: CCAAT-enhancer-binding protein beta; CSF: cerebrospinal fluid; HD: Huntington’s disease; IL: interleukin; MFG-E8: milk fat globule-EGF factor 8 protein; MS: multiple sclerosis; mHTT: mutant Huntingtin; PD: Parkinson’s disease; RRMS: relapsing–remitting MS; RhoA/ROCK2: Ras homolog family member A kinases/Rho-associated kinases 2; SYN: alpha-synuclein; TrkC.T1: Tropomyosin receptor kinase C.T1; TNF-α: tumor necrosis factor alpha; sALS: sporadic ALS; SMAD2: mothers against decapentaplegic homolog 2. ↑: upregulated; ↓: downregulated.

## 9. Conclusions

Numerous studies have described miRNA functions and their aberrant expression affecting neuronal and non-neuronal mechanisms in AD, PD, MS, HD, and ALS. The emerging data also outlined the overlapping functions across the NDs. The extensive research results have improved our knowledge on the remarkable potential value for diagnosis, prognosis, prevention, and treatment of NDs based on up- or downregulated miRNAs expressions. However, there are still significant challenges to surmount since most miRNA-based therapeutic data are on preclinical models, and further studies are needed to increase human safety and efficacy [10,11]. One single miRNA may display several mechanisms and interact with other miRNAs, increasing the complexity of the cellular mechanisms affected in ND, thus leading to unwanted side effects and reducing the efficacy of the treatment. Moreover, miRNA-delivering-improvements are required to efficiently access the target during therapy [11]. To conclude, future identification and characterization of novel miRNAs involved in NDs are highly desired to improve the potential of this novel and up-and-coming research field.

## Figures and Tables

**Table 1:** Profile of miRNAs proposed as Alzheimer’s disease biomarkers.

miRNA	Source	Cohort	Criteria	Target	Alteration	Reference
miR-483-5p	Plasma	40 AD 40 MCI 20 HC	MMSE-FDLA DR-MOCA	Not mentioned	↑	[46]
miR-34a	Plasma	21 + 15 AD 21 + 15 MCI 21 + 15 HC	MMSE	Presynaptic-related protein: VAMP2, SYT1 Antiapoptotic protein: BCL-2	↑	[73]
miR-23	Serum	30 AD 30 HC	MMSE-ROC	Not mentioned	↑	[77]
miR-30b-5p	Blood derived EVs	8 + 40 AD 8 + 40 HC	ROC	Not mentioned	↑	[80]
miR-22-3p				MAPK14		
miR-378a-3p				MAPK14, GOLT1A, PARVA, MAPK1, IGF1R, HDAC4		
miR-212	Brain	5 AD 5 HPC 5 HC	ROC	Not mentioned	↓	[81]
miR-132	CSF	11 AD 7 HPC 9 CT		ITPKB		
	Plasma-derived EVs	16 AD 16 AD-MCI 31 CT				
miR-30a-5p miR-34c miR-27a-3p	CSF EVs	23 + 19 AD 17 MCI HC 18	MMSE	BDNF p53, SIRT1 Not mentioned	↑	[82,84]
miR-146a	Blood	19 progressor MCI 26 stable MCI	MMSE	TLR2, RyanR3	↑	[83]
miR-181a				Fidgetin, BCL-2, SIRT1, RyanR3		

AD: Alzheimer’s disease; BDNF: brain-derived neurotrophic factor; BCL-2: B-cell lymphoma 2; CSF: cerebrospinal fluid; DR: dementia rating; EVs: extracellular vesicles; FDLA: functional daily living activity; GOLT1A: Golgi transport 1A; HC: healthy controls; HPC: high anthropological controls; HDAC4: Histone deacetylase 4; ITPKB: inositol-trisphosphate 3-kinase B; IGF1R: insulin-like growth factor 1; MCI: mild cognitive impairment; MMSE: mini-mental state examination; MAPK14: mitogen-activated protein kinase 14; MOCA: Montreal cognitive assessment; PARVA: Parvin alpha; ROC: receiver operating characteristic; RyanR3: ryanodine receptor 3; SIRT1: sirtuin 1; SYT1: synaptotagmin-1; TLR2: toll-like receptor 2 precursor; VAMP2: vesicle associated membrane protein 2; ↑: upregulated; ↓: downregulated.

**Table 2:** Profile of miRNAs proposed as Parkinson’s disease biomarkers.

miRNA	Source	Cohort	Criteria	Target	Alteration	Reference
miR-150	Serum	80 PD 60 HC	Hoehn-Yahr scale	*AKT3*	↓	[109]
miR-626	CSF	20 PD 27 HC	Hoehn-Yahr stage	Not mentioned	↓	[110]
miR-27b-3p miR-27a-3p	PBMCs	30 PD 14 HC	Hoehn-Yahr stage	*SRRM2*	↑ ↓	[112]
miR-885	PBMCs	36 PD 16 HC	Hoehn-Yahr stage	*IGF1R, CTNNB1, MAN1C1, OXR1*	↑	[118]
miR-17				*E2F1, WEE1, CCND1 - CDKN1A (p21), PTEN, BCL2L11 (BIM), RB1, RBL1 (p107), RBL2 (p130)*	↓	
miR-361				*STAT6. GABPA, BCL6, HIF1A, OXR1*	↓	
miR-26a	CSF	28 PD 4 HC	Hoehn-Yahr stage	DAPK1 protein	↓	[102]
miR-34a-5p	Plasma EV	15 PD 14 HC	UPDRS, Hoehn-Yahr stage, BDI	*D1, SIRT1, BCL-2*	↑	[119]
miR-153 miR-223	Saliva	84 PD 83 HC	UPDRS, Hoehn-Yahr stage	*SNCA, HMOX1*	↓	[120]
miR-30c-2-3p	Plasma EVs	30 PD 30 HC	MDS, Hoehn-Yahr stage	*TNFAIP8L2, NAMPT…*	↑	[121]
miR-15b-5p				*PAX7, SALL1, PTPRR*	↓	
miR-138-5p				*CLMP, KANK1, LMAN1*	↓	
miR-338-3p				*PTEN, FRMD3, ATXN7L*	↓	
miR-106b-3p				*ZNF827*	↓	
miR-431-5p				*CD34, NR3C2, FAM65B*	↓	

BDI: Beck Depression Inventory (evaluation of depression of PD patients); CSF: cerebrospinal fluid; DAPK1: death-associated protein kinase 1; EV: extracellular vesicle; HC: healthy controls; MDS: International Parkinson and Movement Disorder Society; PD: Parkinson’s disease; PBMCs: peripheral blood mononuclear cells; UPDRS: Unified Parkinson’s disease rating scale; ↑: upregulated; ↓: downregulated.

**Table 3:** Profile of miRNAs proposed as multiple sclerosis biomarkers.

miRNA	Source	Cohort	Criteria	Target & Roles	Alteration	Reference
miR-182-5p miR-183-5p	Blood erythrocyte-derived EV	23 MS 22 HC	McDonald, ARMSS, MSSS, EDSS scores	Glossopharyngeal nerve development, Histone H3-K27 demethylation	↑	[142]
miR-128-3p	Serum	74 MS 17 HC	EDSS score	Th1 response p53 Pro-apoptotic pathway	↑	[143,144]
miR-191-5p	Serum	53 RRMS 20 PPMS 27 HC	EDSS score	BDNF expression Neuronal and immune cell apoptosis	↑	[144]
miR-24-3p				BIM PUMA Th1/Th2 balance regulation		
miR-18a-5p	Blood	32 MS 32 HC	Complementary, diagnostic tests	p53 MAPK signaling pathway Apoptosis pathway Th17 cell differentiation	↓	[145]
miR-146a miR-155	Serum	30 MS 30 HC	EDSS score	Th1 and Th17 differentiation	↑	[133]
miR-300	Serum	39 RRMS 35 SPMS 10 HC	McDonald, EDSS	Vasohibin 2 gene Neuron differentiation	↓	[146]
miR-450b-5p				*SOX2* and *PTPRZ1* genes Neuron differentiation and development Neurogenesis regulation		
miR-106a-5p	Blood	32 MS 32 HC	Not mentioned	*RBL2, APP, CYP19A1, BMP2*	↓	[147]
miR-150 miR-328	CSF	86 MS 55 OND	McDonald 2010	Not mentioned	↑	[148]
miR-30a-5p miR-645 miR-21 miR-199a-3p miR-191 miR-365 miR-106a miR-146a					↓	
let-7b-5p	CSF	141 MS 20 HC	McDonald 2010, EDSS	Inflammation Neuronal homeostasis RNA metabolism Anti-Inflammatory Regulator of cytokines, chemokines, growth factors	↓	[149]

ARMSS: age-related multiple sclerosis severity scores; BDNF: brain-derived neurotrophic factor; BIM: Bcl-2-like protein 11; CSF: cerebrospinal fluid; EVs: extracellular vesicles; EDSS: expanded disability status scale; HC: healthy control; MAPK: mitogen-activated protein kinase; MS: multiple sclerosis; MSSS: multiple sclerosis severity scores; PPMS: primary progressive MS; PUMA: p53 upregulated modulator of apoptosis; OND: other neurological diseases; RRMS: relapsing–remitting MS; RNA: Ribonucleic acid; Th17: T-helper 17; ↑: upregulated; ↓: downregulated.

**Table 4:** miRNAs’ expressions and roles in Huntington disease models.

miRNA	Role in HD pathophysiology	Model	Alteration	Reference
miR-128a	Metabolic pathways, particularly cholesterol (affected by mutant HTT)	Human plasma	↑	[168]
miR-122-5p			↓	
miR-140-5p	Regulation of ADAM10 expression	Human CSF	↑	[169,177]
miR-124	Regulator of neuronal differentiation and survival	*STHdhQ111/HdhQ111* cells R6/2 mouse striatum	↓	[170,178]
miR-34a-5p	Neuronal development Brain ageing Metabolic regulation p53/miR-34a/SIRT1 pathway	Brain CAG144 R6/2 mouse	↓	[84,173]
miR-196a	Cytoskeleton modification RANBP10 regulation	HD-iPSCs R6/2 mouse brain RANBP10-R6/2 mouse brain	↓	[175]

ADM10: A disintegrin and metalloproteinase 10; CSF: cerebrospinal fluid; HD: Huntington disease; HTT: huntingtin; iPSCs: induced pluripotent stem cells; RANBP10: RAN binding protein 10; SIRT1: sirtuin1; ↑: upregulated; ↓: downregulated.

**Table 5:** Profile of miRNAs proposed as Huntington’s disease biomarkers.

miRNA	Source	Cohort	Criteria	Target	Regulation	Reference
miR-10b-5p miR-486-5p	Plasma	26 HD, 4 asymptomatic HD 8 HC	Not mentioned	*HTT, BDNF* Not mentioned	↑	[161]
miR-9*	Peripheral leukocytes	36 HD 8 pre-symptomatic HD 28 HC	UHDRS	*HTT,* CoREST	↓	[166]
miR-34b	Plasma	27 HD 12 HC	UHDRS, TFC	*HTT*	↑	[167]
miR-128a	Plasma	15 HD 7 HC	UHDRS, TFC	*HTT, HIP1, SP1…*	↑	[168]
miR-122-5p				*AACS, ADAM10, BCL2…*	↓	
miR-520f-3p miR-135b-3p miR-4317 miR-3928-5p miR-8082 miR-140-5p	CSF	30 Prodromal HD 15 diagnosed HD 10 HC	UHDRS	Not mentioned	↑	[169]

CSF: cerebrospinal fluid; HC: healthy control; HD: Huntington’s disease; CoREST: corepressor of repressor element 1-silencing transcription factor; TFC: total functional capacity; UHDRS: unified Huntington’s disease rating scale; ↑: upregulated; ↓: downregulated.

**Table 6:** Profile of miRNAs proposed as amyotrophic lateral sclerosis disease biomarkers.

miRNA	Source	Cohort	Criteria	Target	Alteration	Reference
miR-129-5p	Blood	27 sALS 25 HC	ALS-FR score	HuD control by *ELAVL4* splicing, translation, localization, and stability of neuronal RNAs are controlled by HuD	↑	[194]
miR-206, miR-151a-5p	Serum	27 ALS 13 HC	ALS-FR score	Not mentioned	↑: mild stage ↓: moderate and severe stages	[198]
miR-133a, miR-199a-5p					↓	
miR-423-3p and 151a-5p					↓ mild and terminal stages	
miR-92a-3p, miR-486-5p	Serum	14 ALS 47 HC	EI score	Nε-hexanoyl lysin (an early phase oxidative stress marker reflects neuronal degeneration)	↑	[199]
miR-10a precursor	Muscle biopsy	12 ALS 11 HC	ALS-FR score	Alsin	↑	[200]
miR-125a-5p + precursor				NF-kB activation (neuro-inflammation)	↑	
miR-1291 precursor				ATXN2 and DCTN1	↑	
miR-1260a-5p				TDP43	↑	
miR-30d precursor				C9orf72 (Other proteins related to ALS pathology)	↓	
miR-181a-5p	CSF	24 sALS 24 HC	EI score	C9orf72	↑	[201]
miR-21-5p miR-15b-5p					↓	

ALS: amyotrophic lateral sclerosis; ALS-FRS: ALS Functional Rating Score; ATXN2: ataxin-2; CSF: cerebrospinal fluid; DCTN1: dynactin subunit 1; EI: EI Escorial revised criteria; HC: healthy controls; HUD: ELAV-like protein 4; NF-kB: nuclear factor kappa B; RNA: Ribonucleic acid; sALS: sporadic ALS; TDP43: TAR DNA-binding protein 43. ↑: upregulated; ↓: downregulated.

## Data Availability

Not applicable.

## Abbreviations

miRNAs: MicroRNAs; NDs: neurodegenerative diseases; CNS: central nervous system; AD: Alzheimer’s disease; HD: Huntington’s disease; ALS: amyotrophic lateral sclerosis; PD: Parkinson’s disease; MS: multiple sclerosis; CSF: cerebrospinal fluid.
